# The Effectiveness of Saireito, a Traditional Japanese Herbal Medicine, in Reducing Postoperative Edema after Acquired Ptosis Surgery: A Prospective Controlled Trial

**DOI:** 10.1155/2018/4742305

**Published:** 2018-06-27

**Authors:** Naoki Morimoto, Natsuko Kakudo, Toshihito Mitsui, Atsuyuki Kuro, Yuko Ueda, Masakatsu Hihara, Yoshihito Tanaka, Yujiro Ozaki, Kenji Suzuki, Kenji Kusumoto

**Affiliations:** ^1^Department of Plastic and Reconstructive Surgery, Kansai Medical University, Japan; ^2^Department of Plastic and Reconstructive Surgery, Kansai Medical University Medical Center, Japan

## Abstract

**Background:**

Persistent edema is a common complication after the surgical treatment of blepharoptosis; however, no objective methods have been established for evaluating or treating this condition. We focused on the Japanese herbal medicine, Saireito, and evaluated its efficacy in reducing postoperative edema.

**Methods:**

This was a prospective, nonrandomized, and controlled study. We evaluated the incidence of postoperative edema in a Control group using a subjective patient-assessed visual analog scales (VASs) to assess swelling, pain, itching, and local warmth and an objective surgeon-assessed VAS to evaluate swelling. Swelling was also assessed by an objective computer-based analysis of digital images. These methods were used to evaluate the effects of Saireito (8.1 g/day) for 8 weeks.

**Results:**

A total of 49 patients and 80 eyelids were enrolled. Twenty-nine patients and 48 eyelids were assigned to the Control group, and 20 patients and 32 eyelids were assigned to the Saireito group. Our analysis of the Control group indicated that postoperative edema persisted for up to 8 weeks. On the other hand, the postoperative edema in the Saireito group was mostly eliminated at 8 weeks. The computer-based analysis of digital images showed that the edema tended to be reduced in the Saireito group compared with the Control group.

**Conclusion:**

Saireito might be effective for reducing postoperative edema after blepharoptosis surgery and almost completely eliminated it at 8 weeks after surgery. This study was a preliminary and nonrandomized study; therefore, the randomized and placebo-controlled study will be needed in the next step.

## 1. Introduction

Mechanical blepharoptosis, such as aponeurotic ptosis and/or dermatochalasis of the upper eyelids, is the most common cause of acquired ptosis [1]. Complications after ptosis surgery, including major complications such as retrobulbar hematoma and ectropion, are rare. On the other hand, minor complications, including subcutaneous hematoma, eyelid malposition, scarring, and persistent edema, remain very common [2, 3]. Edema, which often continues for more than 4 weeks in the clinical setting, causes suffering and sometimes results in eyelid malposition, scarring, or the recurrence of ptosis [2, 3]. Edema within one week is treated or prevented by cooling, compression, or the administration of steroids; however, there are no well-established techniques for the management of persistent edema.

The Japanese herbal medicine Saireito is a combination of two herbal medicines (Shosaikoto and Goreisan) that is used to treat inflammatory diseases as well as autoimmune diseases such as rheumatoid arthritis, cirrhosis, and nephrotic syndrome [4–7]. Saireito has also been demonstrated to be effective in treating edema after total hip arthroplasty and lymphedema after radiotherapy [8, 9]. In addition, it has been reported to have antifibrotic properties; thus, it is useful for the treatment of fibrosis or hypertrophic scars [10, 11].

In the present study, we investigated the incidence of postoperative edema at up to eight weeks after the surgical treatment of ptosis and evaluated the efficacy of Saireito in reducing postoperative edema.

## 2. Methods

### 2.1. Study Design

Each patient in this multicenter, prospective, nonrandomized, controlled study underwent acquired ptosis surgery, which involved the incision of the eyelid fold. This study was performed at Kansai Medical University Hospital and Kansai Medical University Medical Center. All operations performed at the two locations were performed by specialists in plastic and reconstructive surgery. During the study period, there were no established methods for evaluating edema after eyelid ptosis surgery. Thus, in the present study, we used a novel method to evaluate postoperative edema in 30 patients who underwent ptosis surgery but who did not receive Saireito (Control group). Then, after confirming that our evaluation method was reasonable, we evaluated postoperative edema in 30 patients who underwent ptosis surgery and who were treated with Saireito (Saireito group).

The protocol of the Control group study was approved by the institutional review boards of Kansai Medical University Hospital (permit number: H130938) and Kansai Medical University Medical Center (permit number: 25-7). The protocol of the Saireito group study was approved by the institutional review board of Kansai Medical University Hospital (permit number: H140407) and Kansai Medical University Medical Center (permit number: 26-22). The Saireito group study was registered in the UMIN Clinical Trials Registry (number: UMIN000016258). The Control group study was not registered in the UMIN Clinical Trials Registry because it was an observational study without any intervention; thus, at the time of the study, registration was not required according to the Japanese Ethical Guidelines for Medical and Health Research Involving Human Subjects.

### 2.2. Patients

Patients who met the eligibility criteria were recruited for the Control group from September 2013 to September 2014 (Table 1). The patients in the Saireito group were recruited from December 2014 to March 2016; the same eligibility criteria were applied. Patients who received herbal medicine within 2 weeks before surgery were excluded from the study. The target sample size of each group was 30. The baseline data that were collected before enrollment included age, sex, comorbidities, and history of ptosis surgery.

### 2.3. Interventions

Under local anesthesia, the skin along the patient's eyelid fold was incised and the redundant eyelid skin was removed according to the degree of dermatochalasis. The levator aponeurosis was dissected from the tarsus and advanced or resected to the extent that was required. The aponeurosis and skin were sutured using 6-0 or 7-0 nylon. In the Saireito group, Saireito (EK-114, Kracie Pharma, Ltd., Tokyo, Japan) was administered orally as a solution immediately before meals at a dose of 2.7 g three times daily (8.1 g/day), for 8 weeks, starting just after surgery. According to the manufacturer's information, Saireito is a water extract of herbal powder; 8.1 g of Saireito is prepared from a decoction of the following 12 medicinal herbs: Bupleurum root (7.0 g), Pinellia tuber (5.0 g), Alisma rhizome (6.0 g), Scutellaria root (3.0 g), Ginseng radix (3.0 g), Jujube fruit (3.0 g), Poria sclerotium (4.05 g), Polyporus sclerotium (4.5 g), Atractyloides rhizome (4.5 g), Cinnamon bark (3.0 g), Glycyrrhizae root (2.0 g), and Ginger rhizome (1.0 g). Saireito is approved as a herbal medicine under the Japanese National Health Insurance Program and is indicated for the relief of edema [5–10].

### 2.4. The Evaluation of Postoperative Edema after Blepharoptosis Surgery

The subjective symptoms of postoperative edema, including swelling, pain, itching, and local warmth of the affected eyelid, were evaluated using 10-cm visual analog scales (VASs) [12, 13]. Briefly, the item was given a score of 0 to 10 along a continuous 10-cm line, where 0 indicated the absence of symptoms and 10 indicated symptoms of the strongest intensity.

In the present study, we used a high-resolution digital camera system (Robo Skin Analyzer CS 50, NIIC Co. Ltd., Tokyo, Japan) to obtain digital images and evaluate postoperative edema [1]. The images, which were captured under highly uniform brightness [4], were analyzed using the Clinical Suite 2.1 imaging software program (NIIC Co. Ltd.) and shadow mode images were prepared. This mode detects the surface asperity of the facial skin according to the intensity of the reflected light and provides color-coded images in which blue represents the lowest degree of reflection (concave-area) and red represents the highest degree of reflection (convex-area). In this study, we used five-phase color-coded images to indicate the degree of reflection (lowest to highest: blue, green, yellow, orange, and red). We evaluated the percentage of each area of color in each eyelid using the RainbowAnalyze software program (Corefront Co. Ltd., Tokyo, Japan).

The degree of swelling was also objectively evaluated by six blinded plastic and reconstructive surgery specialists at the two hospitals from patient photographs. All surgeons were blinded to the patient information and observed the patient's photos before surgery, just after surgery, and at one, two, four, and eight weeks after surgery; swelling was then evaluated at one and eight weeks after surgery using a 10-cm VAS. Swelling was graded on a scale of 0 to 10 along a continuous 10-cm line, where 0 indicated no symptoms and 10 indicated symptoms of the highest intensity.

### 2.5. Follow-Up

Follow-up examinations were scheduled for one, two, four, and eight weeks after surgery in both groups. Digital images were captured before surgery and at every follow-up examination using a Robo Skin Analyzer. The VAS score was also recorded at every follow-up examination.

### 2.6. Statistical Analysis

The Microsoft Excel software program (Microsoft Office Standard 2010) and the Statcel 3 software add-on (OMS Publishing Inc., Tokyo, Japan) were used for all of the statistical analyses. The age and sex in the Control and Saireito groups were compared using Fisher's exact probability test. The history of ptosis and the internal use of nonsteroidal anti-inflammatory drugs (NSAIDs) in the Control and Saireito groups were compared using a chi-squared test. The VAS scores and the percentage of the colored areas in each group were analyzed using Tukey-Kramer's multiple-comparison post hoc test. The data are presented as the mean + SD (standard deviation). P values of <0.05 were considered to indicate statistical significance.

## 3. Results

**Figure 1:**
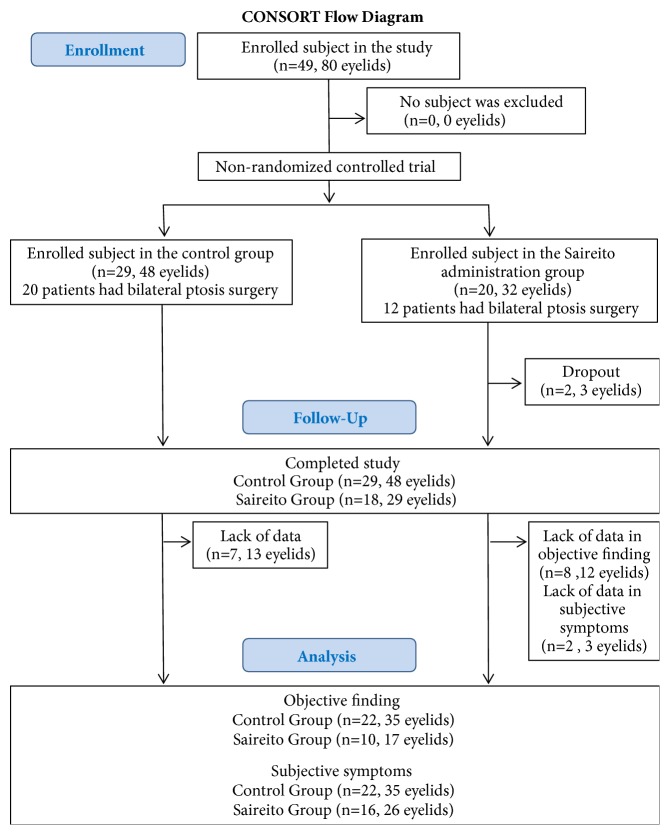
The CONSORT flow diagram.

A total of 49 patients and 80 eyelids were enrolled in the present study (Control group: 29 patients, 48 eyelids; Saireito group: 20 patients, 32 eyelids) (Figure 1). In the Saireito group, one patient chose to discontinue Saireito treatment. Another patient discontinued Saireito treatment due to diarrhea; however, the patient promptly recovered after the discontinuation of Saireito. The data related to the objective findings (digital images analyzed using Robo Skin Analyzer) of 13 eyelids of 7 patients were missing and the subjective data (VAS scales) were also missing in the Control group. Thus, the digital images of 35 eyelids of 22 patients and the VAS scores of 35 eyelids of 22 patients were analyzed in the Control group. The data related to the objective findings of 12 eyelids of 8 patients were missing and the subjective data of 3 eyelids of 2 patients were missing in the Saireito group. Thus, the digital images of 17 eyelids of 10 patients and the VAS scores of 26 eyelids of 16 patients were analyzed. The objective evaluation of swelling of eyelids for which digital images from both 1 and 8 weeks were available (42 eyelids in the Control group; 35 eyelids in the Saireito group) was performed by six surgeons.

The demographic and surgical characteristics of the whole study population are shown in Table 2. The average ages of the patients in the Control and Saireito groups were 73.4 years and 66.5 years, respectively. In the analysis of the objective findings, the average ages of the patients in the Control and Saireito groups were 74.2 years and 65.1 years, respectively. In the analysis of the subjective findings, the average ages of the patients in the Control and Saireito groups were 74.1 years and 65.2 years, respectively. There were no significant differences between the Control and Saireito groups with regard to age or sex (Fisher's exact probability test) or the history of ptosis and the internal use of NSAIDs (chi-squared test) in either the whole study population or the analysis groups of the objective findings and the subjective findings.

**Figure 2:**
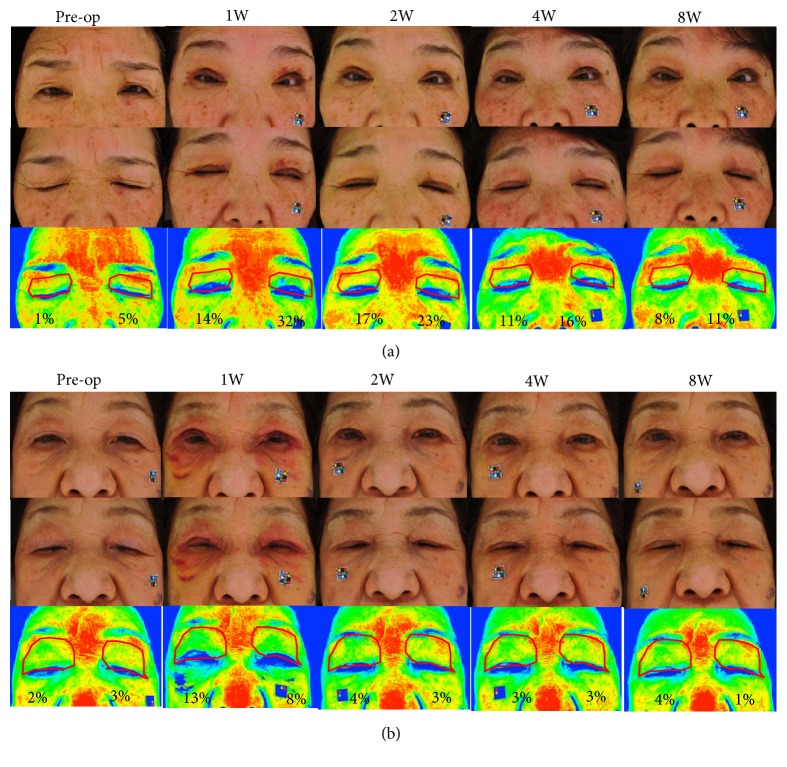
The time course of representative cases in the Control group ((a) a 68-year-old woman with bilateral aponeurotic ptosis) and the Saireito group ((b) a 74-year-old woman with bilateral aponeurotic ptosis). Gross photos were captured with the patient's eyelids open (top) and closed (middle) before surgery and at 1, 2, 4, and 8 weeks after surgery. Color-coded images with the patient's eyes closed are shown at the bottom. The red line indicates the evaluated eyelid area. The percentage indicates the percentage of the blue area in each eyelid.

The time course of representative cases from the Control and the Saireito groups is shown in Figures 2(a) and 2(b), respectively. The representative patient of the Control group showed severe bilateral eyelid edema at 1 week after surgery and mild-to-moderate edema persisted from 4 to 8 weeks after surgery. On the other hand, the patient in the Saireito group developed severe edema at 1 week; however, the edema was mild at 2 weeks and had almost completely regressed at 4 weeks.

**Figure 3:**
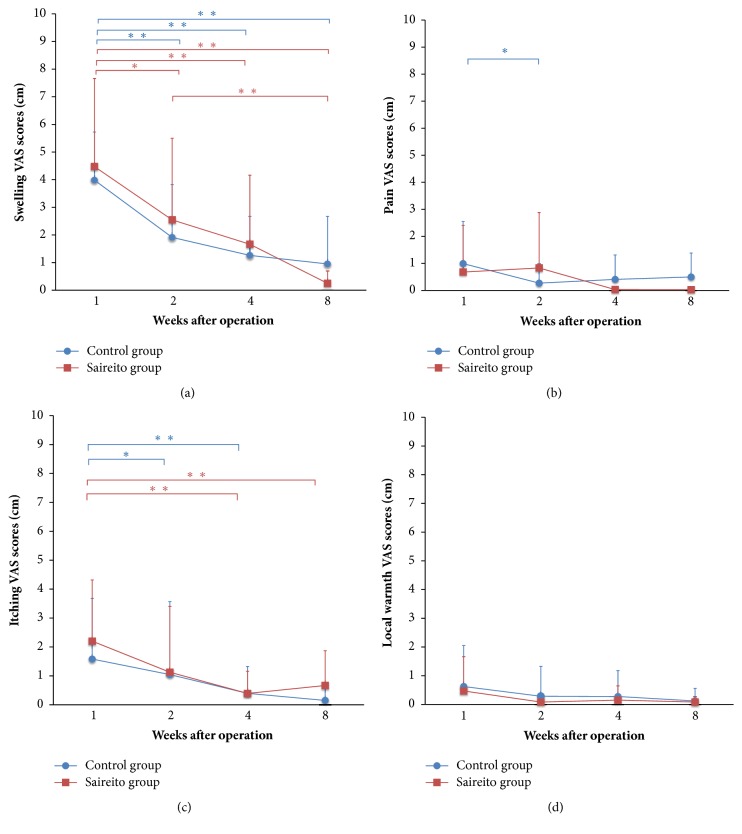
The time course of the subjective VAS scores of swelling (a), pain (b), itching (c), and local warmth (d). (a) In the Control group, the swelling scores at 2 (p<0.01), 4 (p<0.01), and 8 (p<0.01) weeks were decreased in comparison to the score at 1 week. In the Saireito group, the swelling scores at 2 (p<0.05), 4 (p<0.01), and 8 (p<0.01) weeks were decreased in comparison to the score at 1 week. The score at 8 weeks was decreased in comparison to that at 2 weeks (p<0.01). At 8 weeks, the swelling score of the Saireito group was mostly eliminated.  ^*∗∗*^p<0.01,  ^*∗*^p<0.05. (b) In the Control group, the pain score at 2 weeks was decreased in comparison to the score at 1 week (p<0.05). The pain score in the Saireito group was zero at 4 and 8 weeks (p<0.05).  ^*∗*^p<0.05. (c) In the Control group, the itching scores at 2 (p<0.05) and 4 (p<0.01) weeks were decreased in comparison to the score at 1 week. In the Saireito group, the itching scores at 4 (p<0.01) and 8 (p<0.01) weeks were decreased in comparison to the score at 1 week.  ^*∗∗*^p<0.01,  ^*∗*^p<0.05. (d) No significant differences were observed in the local warmth scores.

The results of the analysis of the subjective VAS scores are summarized in Figure 3. The VAS score for swelling decreased with time in both the Control and Saireito groups (Figure 3(a)). Swelling was still present at 8 weeks in the Control group and the swelling had completely disappeared at 8 weeks in the Saireito group. Furthermore, pain had completely disappeared at 4 weeks after surgery in the Saireito group, while some pain was observed in the Control group (Figure 3(b)). The VAS score for itching decreased with time in both the Control and Saireito groups. Curiously, however, the score of the Saireito group was higher than that of the Control group (Figure 3(c)). The VAS for local warmth did not differ between the two groups to a statistically significant extent (Figure 3(d)).

**Figure 4:**
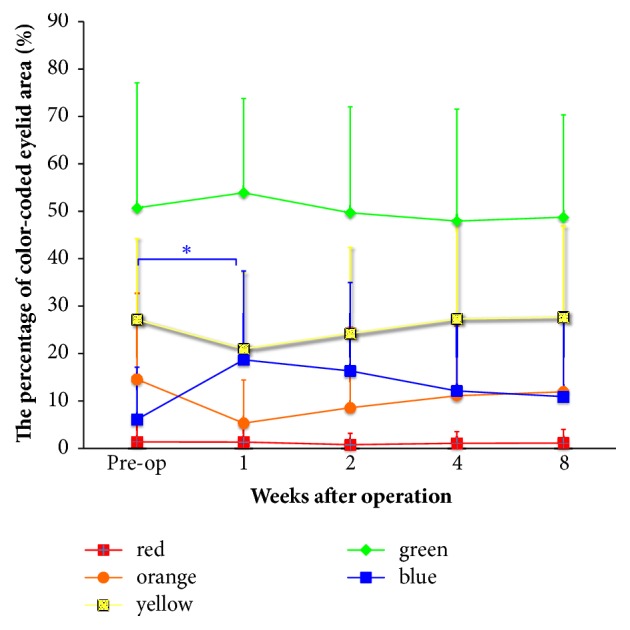
The time course of each color-coded eyelid area in the Control group. The percentages of each color-coded area at preoperation and at 1, 2, 4, and 8 weeks after surgery are shown. The only significant difference that was observed was in the blue color area in the preoperation image and the image from 1 week after surgery (p<0.05).  ^*∗*^p<0.05.

**Figure 5:**
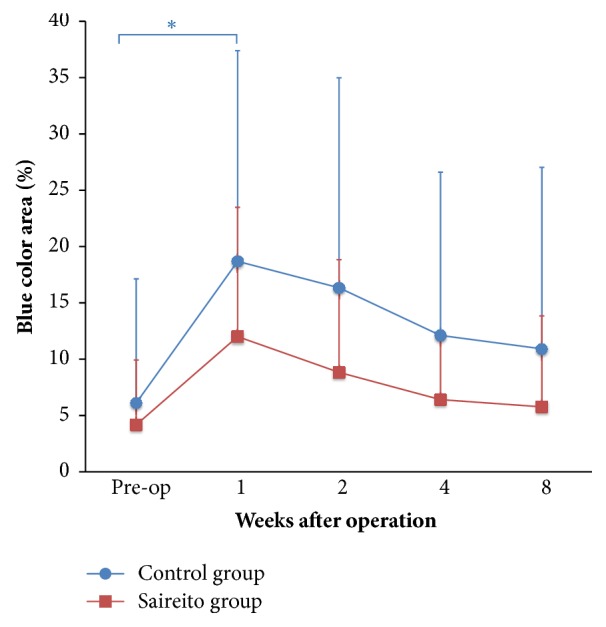
The blue color eyelid area in the Control and Saireito groups. In the Control group, the blue area was significantly increased at 1 week (p<0.05). In the Saireito group, no significant differences were observed during the study period.  ^*∗*^p<0.05.

Next, we evaluated the time course of the changes in each color area in the color-coded images of the Control group to determine an objective method for evaluating swelling. We evaluated color-coded images that were captured with the eyelids closed because the eyelid area was too small to evaluate when the eyelids were open. No significant differences were observed in the red, orange, yellow, or green colors; however, the blue area at 1 week after surgery was significantly increased in comparison to that in the Control group before surgery (Figure 4). In the Saireito group, the blue area—which reflects the concave surface along the eyelid fold—was not increased at 1 week and the blue area in the Saireito group tended to be reduced after surgery in comparison to the Control group (Figure 5).

**Figure 6:**
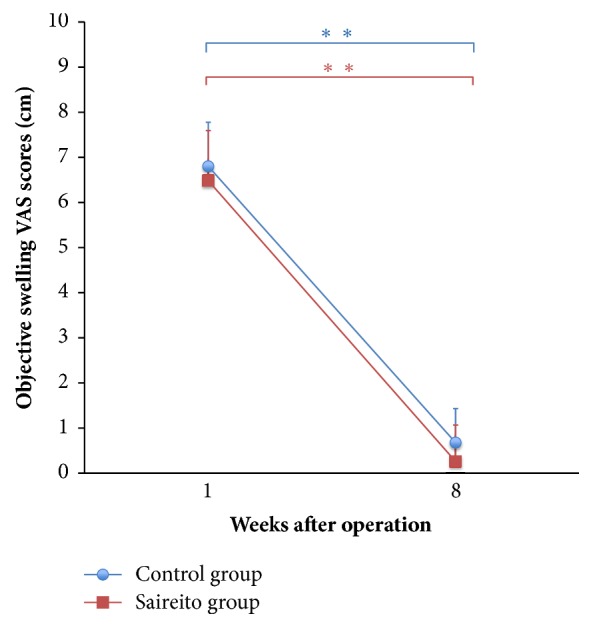
The VAS scores for objective swelling. In both groups, the scores at 8 weeks were significantly decreased in comparison to the scores at 1 week (p<0.01).  ^*∗∗*^p<0.01.

The objective swelling scores of the Control group and the Saireito group at 8 weeks were decreased in comparison to the score at 1 week (Figure 6).

## 4. Discussion

Persistent postoperative edema is a common complication after ptosis surgery; however, an objective method for evaluating edema following eyelid ptosis surgery has not been established and no methods for objectively evaluating its duration have been reported. Studies using 3D imaging systems to evaluate persistence of postoperative edema following orthognathic surgery have revealed that 10–20 percent of primary edema remained at 3 months after surgery and that primary edema had almost disappeared at 6 months after surgery [14, 15].

In the present study, we evaluated postoperative edema after ptosis surgery using the subjective patient-assessed VAS scores, objective surgeon-assessed VAS scores, and the computer-based analysis of images captured using a digital camera. Ptosis surgery is a basic operation. The plastic and reconstructive surgery specialists in our hospital used the same procedure when treating patients with ptosis.

Postoperative edema of the head and neck region has been evaluated according to the thickness of the soft tissue measured by ultrasound, cone beam computer tomography, and magnetic resonance imaging [16–18]. However, these methods are time-consuming for postoperative outpatients. The three-dimensional evaluation of the postoperative swelling using a 3D Digitizer is another method [19, 20]; however, the eyelids should not be exposed to laser light, as it may cause visual impairment. Our analysis of the Control group indicated that postoperative edema persisted—to some extent—at 8 weeks after surgery. We performed a computer-based analysis of images captured using a digital camera to evaluate edema because it was rapid and noninvasive method. We initially expected the red or orange area to increase after surgery because the edematous area is brightly reflected and is indicated by red or orange after surgery. Unexpectedly, however, these areas did not increase and the blue color area significantly increased. This was probably because the eyelid fold is sutured and fixed to the tarsus and is largely depressed in the edematous eyelid after ptosis surgery (Figure 4). The most severe edema was observed at 1 week; the subjective VAS scores indicated its gradual improvement. The depressed blue area changed to reflect the degree of edema in the same way. Thus, our image analysis reflected the state of eyelid edema well and may be used to evaluate eyelid edema.

With regard to the effectiveness of Saireito, the subjective VAS scores of edema and pain and the objective VAS scores at 8 weeks (with the exception of itching) indicated that a certain level of postoperative edema persisted for up to 8 weeks in the Control group. The scores revealed that little or no edema occurred in the Saireito group at 8 weeks. The blue area increased in the Control group at 1 week; in contrast, no significant change in the blue area in the Saireito group was observed during the study period. These results suggest that the administration of Saireito might reduce the incidence of postoperative edema to some extent and that it had almost eliminated postoperative edema at 8 weeks. The only adverse event observed in this study was mild diarrhea; no severe adverse events occurred in association with the administration of Saireito [5, 8–10].

With regard to other anti-inflammatory drugs, steroids can be administrated once or for a few days after surgery and have been reported to reduce edema within several days [20, 21]. The administration of NSAIDs for several months after surgery may cause gastrointestinal ulceration; thus, it is not implemented in clinical practice [22]. Hilotherapy is a novel cooling method that is used to reduce perioperative pain and swelling in facial surgery; however, this method can only be used for a few days after surgery [12]. It is suggested that the safety of Saireito might be superior to these conventional therapies when it is administered for a number of weeks.

This study was a preliminary and nonrandomized study; therefore, the randomized and placebo-controlled study will be needed in the next step to show the efficacy of Saireito clearly.

## 5. Conclusion

Saireito, a traditional Japanese herbal medicine, seemed to be effective for reducing postoperative edema after acquired ptosis surgery and had almost eliminated postoperative edema at 8 weeks after surgery.

## Figures and Tables

**Table 1:** Inclusion and exclusion criteria.

**Inclusion criteria**
(i) Age ≥20 years
(ii) Scheduled to undergo ptosis surgery
**Exclusion criteria**
(i) The use of oral herbal medicine within 2 weeks before surgery
(ii) Any of the following systemic diseases or conditions:
(a) The continuous use of oral corticosteroid therapy (>5 mg/day prednisolone equivalent) or an oral immune-suppressing drug
(b) Malignant tumor
(c) Hepatic failure or renal failure
(iii) Judged by the investigator to be inappropriate as the study subject

**Table 2:** The demographics and surgical characteristics.

	Control group	Saireito group
Eyelids	29 (n=48)	20 (n=32)

Age	73.4±7.6	66.5±9.6

Sex (male:female)	14:15	6:14

History of ptosis surgery		
Absence	23	18
Presence	4	0
Unknown	2	2

Comorbidities	High blood pressure (n=5)	High blood pressure (n=5)
(Number of patients,	Diabetes (n=1)	Diabetes (n=2)
multiple selection)	Cataract (n=1)	Glaucoma (n=1)
	Chalazion (n=1)	Rheumatoid arthritis (n=1)
	Meningioma (n=1)	Diabetic retinopathy (n=1)
	Prostatic hypertrophy (n=1)	Bronchial asthma (n=1)
	Atrial fibrillation (n=1)	Hyperlipidemia (n=1)
	Heart disease (n=1)	Reflux esophagitis (n=1)
		Dry eye (n=1)
		Cataract (n=1)
		Suspected cerebral infarction (n=1)
